# Electroacupuncture Downregulating Neuronal Ferroptosis in MCAO/R Rats by Activating Nrf2/SLC7A11/GPX4 Axis

**DOI:** 10.1007/s11064-024-04185-x

**Published:** 2024-05-31

**Authors:** Wei Zhu, Jianjian Dong, Yongsheng Han

**Affiliations:** 1 Institute of Neurology, Anhui University of Chinese Medicine, Hefei, Anhui China; 2 Center for Xin’an Medicine and Modernization of Traditional Chinese Medicine of IHM, Anhui University of Chinese Medicine, Hefei, Anhui China; 3 Wannan Medical College, Wuhu, Anhui China

**Keywords:** Electroacupuncture, Ischemic stroke, Cerebral ischemia/Reperfusion, Nrf2, Ferroptosis, Lipid peroxidation

## Abstract

Ischemic stroke involves various pathological processes, among which ferroptosis is crucial. Previous studies by our group have indicated that electroacupuncture (EA) mitigates ferroptosis after ischemic stroke; however, the precise mechanism underlying this effect remains unclear. In the present study, we developed a rat model of middle cerebral artery occlusion/reperfusion. We chose the main acupoint of the treatment methods of the “Awakening and Opening of the Brain”. Rats’ neurological function and motor coordination were evaluated by neurological function score and the rotarod test, respectively, and the volume of cerebral infarction was analyzed by 2,3,5-triphenyltetrazolium chloride Staining. The cerebrovascular conditions were visualized by time-of-flight magentic resonance angiography. In addition, we detected changes in lipid peroxidation and endogenous antioxidant activity by measuring the malondialdehyde, glutathione, superoxide dismutase activities, glutathione/oxidized glutathione and reduced nicotinamide adenine dinucleotide phosphate/oxidized nicotinamide adenine dinucleotide phosphate ratios. Inductively coupled plasma-mass spectrometry, western blot, reverse transcription-polymerase chain reaction, fluoro-jade B staining, immunofluorescence analysis, and transmission electron microscopy were utilized to examine the influence of EA. The results indicate that EA treatment was effective in reversing neurological impairment, neuronal damage, and protecting mitochondrial morphology and decreasing the cerebral infarct volume in the middle cerebral artery occlusion/reperfusion rat model. EA reduced iron levels, inhibited lipid peroxidation, increased endogenous antioxidant activity, modulated the expression of several ferroptosis-related proteins, and promoted nuclear factor-E2-related factor 2 (Nrf2) nuclear translocation. However, the protective effect of EA was hindered by the Nrf2 inhibitor ML385. These findings suggest that EA can suppress ferroptosis and decrease damage caused by cerebral ischemia/reperfusion by activating Nrf2 and increasing the protein expression of solute carrier family 7 member 11 and glutathione peroxidase 4.

## Introduction

Ischemic stroke (IS) is a medical condition characterized by the interruption of blood supply to the brain due to different factors. This interruption causes localized ischemia and hypoxic necrosis of brain tissues, resulting in neurological deficits. IS is the most common form of stroke, as stated in references [1, 2]. Prolonging the recanalization time window as much as possible and minimizing adverse effects after recanalization are important goals for treating IS [3–5]. Neuronal harm and cell death occur as a consequence of insufficient oxygen and blood circulation following an IS. When blood flow is restored, brain tissue damage and metabolic dysfunction are further aggravated due to reperfusion. Reperfusion injury involves various intricate pathophysiological processes, such as oxidative stress, excessive calcium accumulation, heightened inflammatory response, elevated discharge of excitatory amino acids, apoptosis, and necrosis, among other factors [6, 7]. None of these mechanisms, however, fully explain the brain damage caused by IS.

Ferroptosis, an innovative nonapoptotic type of programmed cellular demise, is distinguished by the division of mitochondria, a heightened density of membranes, a decrease in the quantity or absence of cristae, the buildup of intracellular lipid reactive oxygen species (ROS), and an augmented iron concentration [8, 9]. Several research has indicated that ferroptosis is a primary type of abnormal cell demise following a stroke, and inhibiting ferroptosis has been proven effective in reducing the damage caused by a stroke [10–14]. Tuo et al. [15] found that Ferrostatin-1, an inhibitor of ferroptosis, exerts neuroprotective effects by blocking cystine transport and inhibiting glutathione (GSH) depletion in mouse middle cerebral artery occlusion model. In addition, it was found that antagonizing ferroptosis by enhancing the activity of glutathione peroxidase 4 (GPX4) effectively improved the neurological function of mice with ischemia/reperfusion in the middle cerebral artery, whereas the prognosis of stroke was worsened due to increased ferroptosis after weakening GPX4 function [16]. GPX4 has a pivotal function in regulating ferroptosis and is necessary for the conversion of lipid hydroperoxides into harmless lipids [17–20]. The presence of GPX4 and its essential cofactor GSH is vital for the removal of lipid peroxides. The accumulation of lipid peroxides occurs when GPX4 is inactivated or GSH is depleted, ultimately causing ferroptosis [20, 21]. Furthermore, the malfunction of the cystine/glutamate antiporter (system x_c_^−^) leads to the occurrence of ferroptosis [22]. Blocking the solute carrier family 7 member 11 (SLC7A11) subunit of system xc- leads to a decrease in both GSH levels and GPX4 activity. As a result, lipid peroxide accumulation occurs, thus inducing ferroptosis [23]. Nearly all antioxidant response elements are transcriptionally regulated by nuclear factor-E2-related factor 2 (Nrf2) [24]. Moreover, it promotes the expression of downstream antioxidant enzymes such as GPX4 and heme oxygenase-1 [25]. Nrf2 is an essential transcription factor that regulates cellular redox homeostasis and inflammation [26, 27], controlling SLC7A11 and GPX4 protein expression [24]. Increasingly studies show that demonstrated that ferroptosis is an essential player in ischemic brain injury and that preventing ferroptosis has the potential to protect ischemic brain tissue [28].

Electroacupuncture (EA) is a hybrid form of acupuncture that combines traditional practices with modern electrical stimulation. Its effectiveness lies in its ability to mitigate neuronal damage resulting from cerebral ischemia while simultaneously promoting the restoration of neurological function. This is achieved through various effects, including inhibition of apoptosis, oxidative stress, and inflammation inhibition; autophagy modulation; and neurovascular unit repair [29, 30]. The main acupoints of “Awakening and Opening of the Brain” acupuncture method selected for the experiment is the acupuncture method proposed by academician Shi Xuemin of Tianjin University of Traditional Chinese Medicine for the basic mechanism of stroke [31]. The summarized results of 9005 cases of stroke patients treated with this therapy showed that the total effective rate reached 98.56% [32]. The EA therapy for IS is multifactorial, implicating an intricate interplay among different elements, and EA’s specific mechanism of action remains unclear. Our previous study demonstrated a protective effect of EA in the rat middle cerebral artery occlusion model model and oxygen glucose deprivation/reoxygenation induced neuronal ferroptosis [33, 34]. Expanding on prior research, this study aims to ascertain EA’s protective effect against cerebral ischemia/reperfusion injury and examine whether its effect relates to its impact on ferroptosis and the Nrf2 signaling axis.

## Materials and Methods

### Animals

Eighty-four adult male Sprague-Dawley rats weighing 250–280 g and aged 2–3 months, which were specific pathogen-free (SPF), were purchased from Pizhou Dongfang Experimental Animal Breeding Co., Ltd (license number SCXK2017-0003). All rats were housed under standard laboratory conditions; the temperature was maintained at 24–26℃ and the relative humidity was kept between 50% and 60%. Proper ventilation was ensured, and the rats were housed on a 12-hour light and 12-hour dark cycle. The rats were given free access to food and water. After 7 days of acclimatization, the experiments were conducted.

### Middle Cerebral Artery Occlusion/Reperfusion (MCAO/R) Rat Model and Grouping

**Fig. 1:**
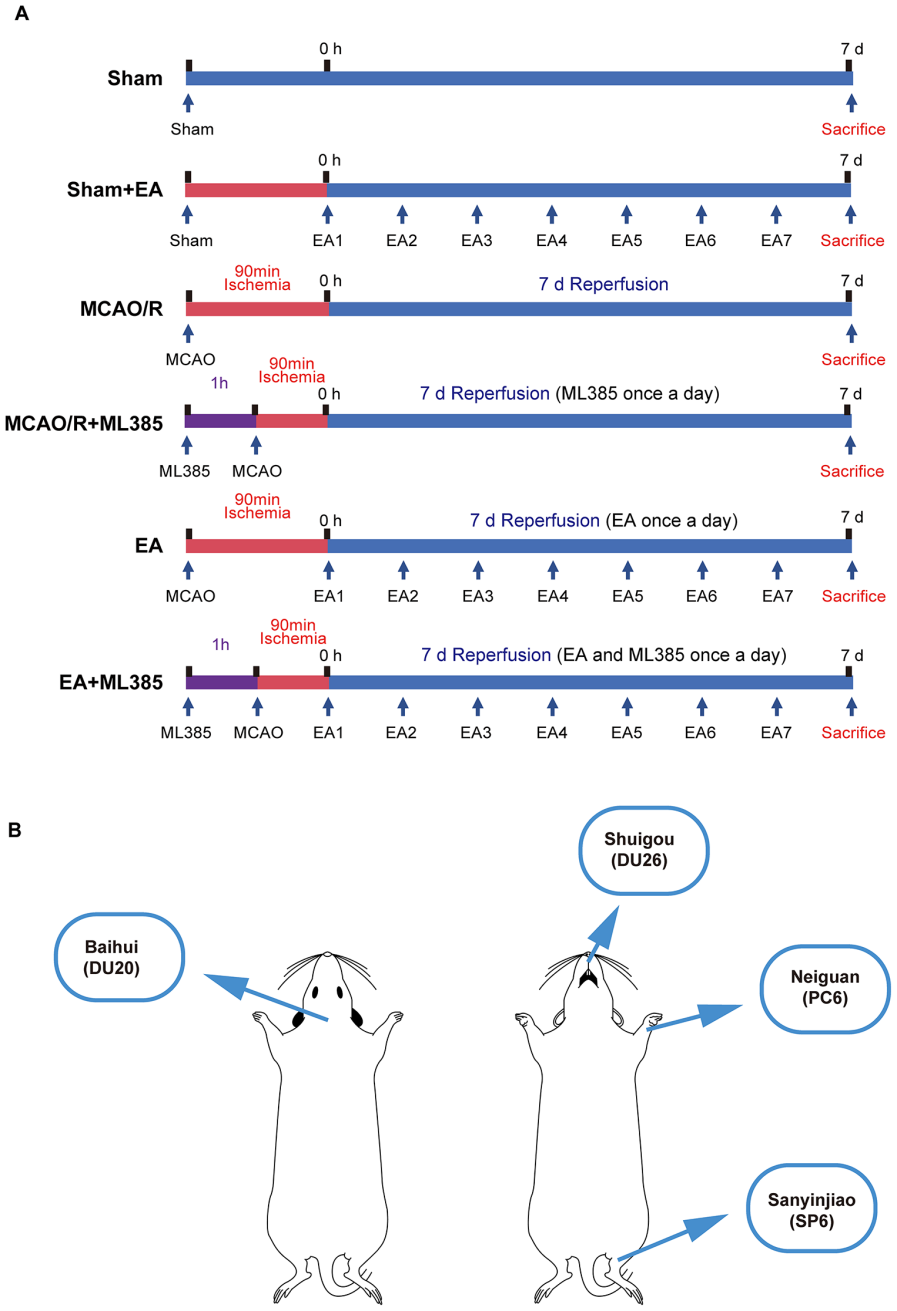
Diagram of experimental procedures and the acupoints used. (**A**) Diagram of experimental procedures. (**B**) Diagram of the acupoint used. Baihui(DU20) is located in the center of the parietal bone in rats, Shuigou(DU26) is located 1 mm below the tip of the rat’s cleft lip in the center of the nose. Neiguan(PC6) is located on the medial side of the forelimb of the rat, about 3 mm away from the carpal joint between the radial and ulnar suture. Sanyinjiao(SP6) is located about 10 mm straight up from the tip of the inner ankle of the hind limb of the rat

The rats were randomly divided into 6 groups: Sham group (apart from nylon filament ligation, all surgical procedures were performed), Sham + EA group (sham operation followed by electroacupuncture), MCAO/R group (MCAO/R model), MCAO/R + ML385 group (MCAO/R model rats with Nrf2 inhibitor ML385), EA group (MCAO/R model rats treated with EA), EA + ML385 group (MCAO/R model rats treated with EA and ML385). To preliminarily assess the effects of EA and ML385, all 6 experimental groups underwent neurological function scoring and TTC staining. Subsequent experiments were performed with the remaining 4 groups except the Sham + EA and MCAO/R + ML385 groups. (Fig. 1A).

Prior to surgery, all rats were fasted for 12 h. The rat model of cerebral ischemia‒reperfusion injury was constructed using middle cerebral artery occlusion, referencing the method reported by Zea Longa [35]. Specifically, the left middle cerebral artery was occluded in all rats. Rats were anesthetized by an intraperitoneal injection of 3% pentobarbital sodium (30 mg/kg) and placed in the supine position with a heating pad to maintain body temperature at 37℃±0.5℃. A minor cut was made in the external carotid artery, and a nylon stitch (A5-2036, Beijing Xinong Technology Co., Ltd.) measuring 0.36 ± 0.02 mm in diameter with a silicone-coated end was inserted, blocking the blood supply of the middle cerebral artery. The suture was slowly withdrawn after 90 min. The neck muscles and skin of the rats were sutured layer by layer and disinfected with iodophor. Once the rats regained consciousness, their neurological function was assessed using modified Longa’s score (0–4) [35] as follows: 0, no noticeable neurological deficits; 1, contralateral forelimb flexion; 2, circling toward the paralyzed side when walking; 3, leaning toward the side opposite the lesion; and 4, inability to walk spontaneously. A score between 1 and 3 indicated successful modeling.

### EA Treatment and Drug Dosage and Administration

The sham group and the MCAO/R group were subjected to grasping under the same conditions as the other group but did not receive electroacupuncture treatment. For the Sham + EA group, EA group and the EA + ML385 group, acupoints were selected based on the rat acupoint atlas established by Li et al. [36]; specifically, the acupoints used for the “Awakening and Opening the Brain” method, which includes the bilateral “Neiguan” acupoints (PC6, pericardium meridian of hand jueyin), “Shuigou” acupoint (DU26, governor vessel), bilateral “Sanyinjiao” acupoints (SP6, spleen meridian of foot taiyin), and “Baihui” acupoint (DU20, governor vessel)(Fig. 1B). Disposable acupuncture needles were used. Following this, electrical stimulation was applied for 30 min for 7 consecutive days using a continuous wave of 2/100 Hz, ~ 2–4 V and 0.5 ~ 1.5 mA. Group EA + ML385 rats were modeled and injected with the Nrf2 inhibitor ML385 (ML385, 30 mg/kg, SJ-MX0153, Sparkjade, Shandong, China) [37, 38] intraperitoneally one hour before modeling daily electroacupuncture treatment.

### Rotarod Test

The rats were tested three times a day for three days, with each trial lasting 5 min and a minimum interval of 30 min between each experiment. Acclimatization was performed on the first two days. The rotating bar speed gradually increased from 5 to 40 rpm in each trial. The latency of each rat to fall off the rotating rod was recorded, and the average value over three experiments was used for analysis.

### Magentic Resonance Angiography (MRA)

The rats were anesthetized with 3.5% isoflurane and oxygen. A respiratory monitoring system was used to monitor vital signs. Image scanning was performed using the Agilent technology 9.4-T/400-mm animal scanner (Agilent Technologies, Santa Clara, CA, USA), and magentic resonance angiography were captured using 3D time-of-flight.

### 2, 3, 5-Triphenyl Tetrazolium Chloride (TTC) Staining

Rats were anesthetized and their brains were removed immediately. Fresh brains were then placed in a -20℃ freezer and quickly frozen for 10 min. The brains were placed in a brain mold and manually sliced coronally. The slices, approximately 2 mm thick, were cut into 5 consecutive coronal sections, ensuring a neat cut surface. These sections were then put in TTC solution preheated to 37℃ and incubated in a 37℃ water bath in the dark for 30 min. Every 10 min, the tissues were gently shaken, and the brain slices were flipped to allow even contact with the staining solution, and the staining results were observed. Once the tissues were stained, they were placed in 4% paraformaldehyde for fixation in the dark for 24 h. After organizing the brain slices in sequence, photographs were captured using a camera. The software ImageJ was used to compute the infarct volume for each rat.

## Fluoro-Jade B (FJB) Staining

Sections were deparaffinized in water and circled using a histology pen. Using freshly prepared 50% glacial acetic acid as the solvent, FJB was diluted 1:400 to prepare a working solution. FJB was added and incubated overnight at 4℃. The nuclei were stained with DAPI for 8 min, and then the sections were rinsed with pure water. After air-drying, the sections were cleared with xylene for 1 min and sealed with rapid-drying mounting medium. Observations of the sections were conducted under a microscope, and images were captured at specific excitation and emission wavelengths for DAPI and the green fluorescent probe.

### Immunofluorescence Staining

The rats were perfused with paraformaldehyde after anesthesia and the brains were immediately removed [39]. Paraffin sections were deparaffinized and then underwent gradient dehydration. After antigen retrieval, the sections were allowed to cool naturally. The slides were washed in PBS on a shaker three times for 5 min each. Blocking serum was then applied, and the sections were incubated in a humidified chamber at 37℃ for 30 min. An anti-Nrf2 primary antibody was added, and the sections were placed in a humidified chamber and incubated overnight at 4℃. The slides were then washed in PBS on a shaker three times for 5 min each. A corresponding secondary antibody was added and incubated at room temperature in the dark for 50 min. The cell nuclei were counterstained with DAPI, followed by another washing step. The slides were then mounted, and Nrf2 expression levels were observed using an optical microscope (C1, Nikon Eclipse).

### Transmission Electron Microscopy (TEM)

After euthanizing the rats, their brains were immediately extracted within 1–3 minutes, taking care to minimize mechanical damage [33]. The extracted tissues were immediately placed into a petri dish and quickly cut into 1 mm^3^ pieces in a dish containing fixative. These smaller tissue blocks were then transferred to an EP tube filled with fresh TEM fixative for further fixation at 4℃. Postfixation was achieved for 2 h in a 1% osmium tetroxide solution, followed by dehydration in graded ethanol solutions. They were then soaked in acetone and embedded in epoxy resin. The embedded samples were placed in a 60℃ oven for polymerization for 48 h. After polymerization, the resin blocks were extracted and set aside. Ultrathin sections of 60–80 nm were prepared from these blocks using an ultramicrotome and mounted on 150-mesh copper grids with a formvar film. The grids underwent staining, were dried overnight at room temperature, and then were examined under a TEM for image acquisition and analysis.

### Measurement of Antioxidant Activity

Rats were anesthetized and their brains were removed immediately. The regulatory effect of EA on ferroptosis was confirmed by measuring the levels of malondialdehyde (MDA), glutathione (GSH), oxidized glutathione (GSSG), superoxide dismutase (SOD) activities and reduced nicotinamide adenine dinucleotide phosphate/ oxidized nicotinamide adenine dinucleotide phosphate (NADPH/NADP+) ratios. The supernatants were collected for analysis after the brain tissue was mechanically homogenized. The MDA and GSH levels, GSH/GSSG ratio, and SOD levels in brain tissues were evaluated utilizing an MDA assay kit (BC0025, Solarbio, Beijing, China), GSH assay kit (BC1175, Solarbio, Beijing, China), GSSG assay kit (BC1185, Solarbio, Beijing, China), and SOD assay kit (BC5165, Solarbio, Beijing, China). The NADPH/NADP + ratio was determined using an NADPH/NADP + assay kit (S0179, Beyotime, Shanghai, China).

### Measurement of Iron Levels by Inductively Coupled Plasma-Mass Spectrometry (ICP‒MS)

Brain tissues (200 mg) were taken from rats in the different groups. The blood on the tissue surface underwent multiple PBS washes, followed by the absorption of moisture on the tissue surface with paper filters. Brain tissues were placed in a magnetic microwave digestion tube, and 6 ml of concentrated ultrapure nitric acid was added. Predigestion was carried out at 130℃ for 30 min, after which the samples were transferred to a microwave digestion tank. The parameters were as follows: the temperature was raised to 300℃ over 10 min and maintained there for 5 min; then, it was raised to 600℃ over another 10 min and held there for 20 min, after which it was gradually decreased to 70℃. The samples were then transferred to a constant-temperature metal bath, and acid digestion was performed at 150℃ for 1 h. Once the solution appeared clear and transparent, it was allowed to cool and reach the temperature of the room. The mixture was then dissolved in ultrapure water to 10 ml and centrifuged at 3000 rpm for 5 min; the supernatant was collected, while the precipitate was discarded. The iron content was measured using ICP‒MS (NexION 350D, PerkinElmer, USA), and the samples were analyzed using the external standard method.

### Reverse Transcription - Polymerase Chain Reaction (RT‒qPCR)

According to the kit instructions (AC0202, Sparkjade, Jinan, China), total RNA was extracted. And according to the instructions of a kit (AG0304, Sparkjade, Jinan, China), 2 µL of the RNA template to be tested was mixed with 1 µL of gDNA Eraser and RNase Free H2O to a volume of 10 µL for reverse transcription. After incubation at 42℃ for 2 min, 10 µL of 2× SPARKscript II RT Plus Master Mix was added so that the total volume was 20 µL. After incubation at 50℃ for 15 min, the samples are heated to 85℃ for 5 s. The obtained cDNA was then prepared for PCR using the ROX Reference Dye II calibration method according to the instructions of a kit (AH0104, Sparkjade, Jinan, China). GAPDH was used as an internal reference to measure the mRNA levels of Nrf2. Amplification was performed with a fluorescent quantitative PCR instrument (CFX Connect, Laboratories, Hercules, USA). The amplification conditions were as follows: predenaturation at 94℃ for 3 min → denaturation at 94℃ for 10 s → annealing/extension at 60℃ for 34 s (40 cycles of denaturation, annealing, and extension). The analysis utilized the Relative Quantification Analysis technique and computed the target gene’s relative expression level by means of the 2^−△△Ct^ method. The primers used are listed in Table 1.

**Table 1:** Primers used for real-time PCR

Genes (rat)	Forward primer (5′-3′)	Reverse primer (5′-3′)
Nrf2	AATTGCCACCGCCAGGACT	TCAAACACTTCTCGACTTACCCC
GAPDH	CTGGAGAAACCTGCCAAGTATG	GGTGGAAGAATGGGAGTTGCT

### Western Blotting (WB)

Total protein was extracted from rat brain tissue by lysis with RIPA tissue lysis buffer. Approximately 20–30 µg of protein was separated on a 10–15% SDS polyacrylamide gel. The protein was then transferred to a 0.2 μm NC membrane. The membrane was blocked with blocking buffer, and primary antibodies, including anti-Nrf2 (1:1000, GB113808, Servicebio, Wuhan, China), anti-SLC7A11 (1:1000, 382,036, Zenbio, Chengdu, China), anti-GPX4 (1:800, 381,958, Zenbio, Chengdu, China), anti-β-tubulin (1:3000, 380,628, Zenbio, Chengdu, China), anti-GAPDH (1:2000, GB11002, Servicebio, Wuhan, China), and anti-histone H3 (1:1000, 381,432, Zenbio, Chengdu, China), were added and overnight incubation at 4℃. The membrane was incubated with a secondary antibody (1:10000, goat anti-rabbit IgG, 511,203, Zenbio, Chengdu, China) after four to five washes at 25 ± 1℃ in 2% BSA/TBS-T for 1–2 h. The bands were visualized using ECL, and the ImageJ software was used to measure the grayscale values of the bands.

### Statistical Analysis

Data analysis was conducted using SPSS 23.0 (IBM, Armonk, New York, USA), and data visualization was performed with GraphPad Prism version 9.5.0 (GraphPad Software, San Diego, California, USA). The data is presented as the mean ± SD. Comparison of the data of each group was analyzed by one-way analysis of variance (ANOVA) and multiple comparison test, and *P* < 0.05 was regarded as statistically significant difference.

## Results

### EA Treatment Ameliorated Neurological Deficits and Reduced the Infarct Volume after MCAO/R

**Fig. 2:**
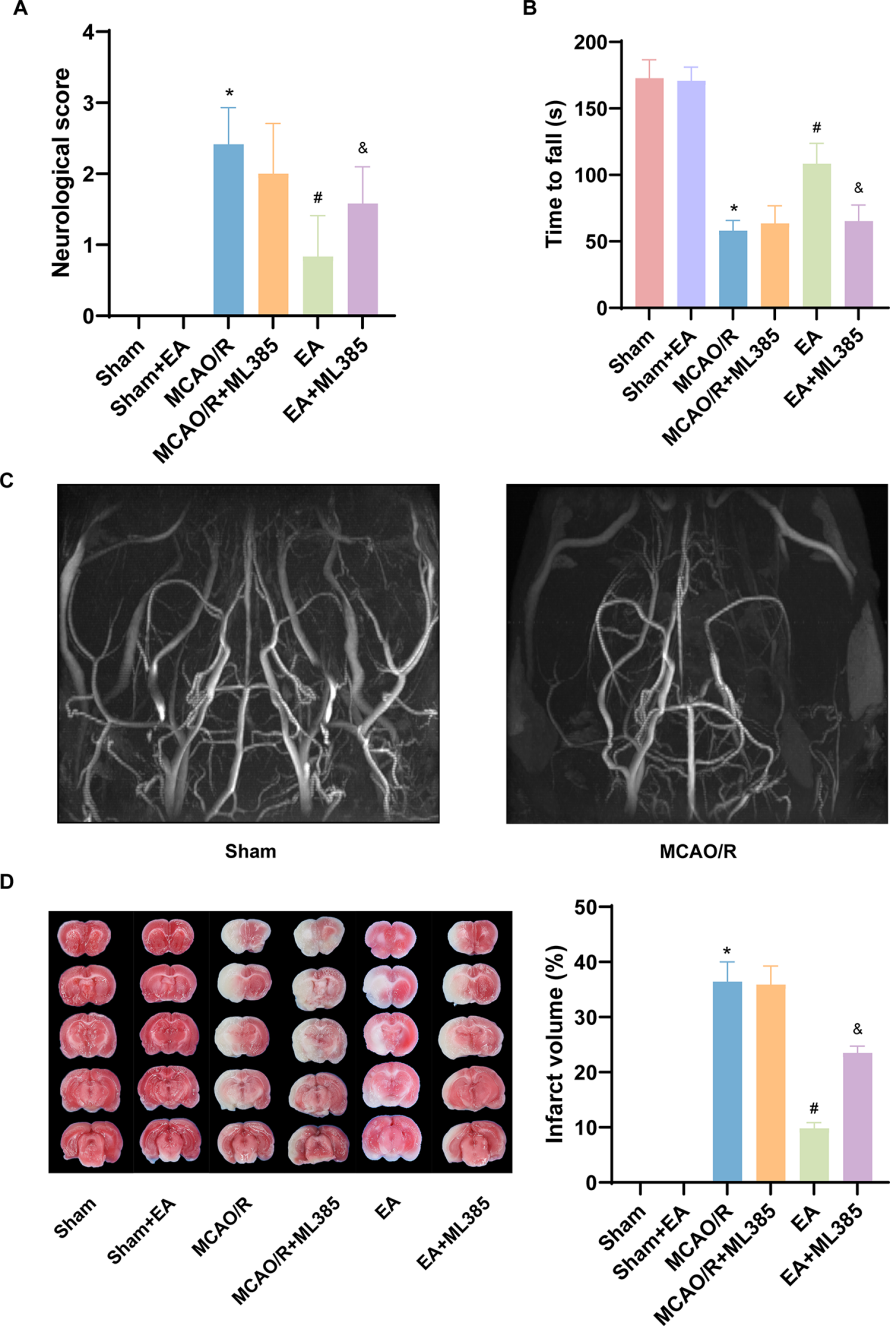
EA treatment attenuated neurological deficit and the infarct volume after MCAO/R. (**A**) Neurological score (*n* = 12). (**B**) Rotarod test results (*n* = 12). (**C**) Magnetic resonance angiography by TOF (Scale bar, 2 mm). (**D**) The infarct volume was measured by TTC staining (*n* = 6). The results are expressed as means ± SD. *p < 0.05 vs. The sham group; #*p* < 0.05 vs. The MCAO/R group; &*p* < 0.05 vs. the EA group

On the 7th day post-surgery, the neurological deficits of SD rats were assessed using Longa’s method and the rotarod test, and the infarct volume was measured. As shown in Fig. 2, the MCAO/R group showed a significant increase in neurological function score and infarct volume and a decrease in latency to fall in the rotarod test compared to the sham group (*p* < 0.05). In comparison to the MCAO/R group, the EA treatment group exhibited a significantly decreased neurological function score and infarct volume and increased latency to fall in the rotarod test (*p* < 0.05). In comparison to the EA group, the EA + ML385 group, showed an increase in the neurological function score (*p* < 0.05) and infarct volume (*p* < 0.05) and a decrease in the latency to fall in the rotarod test (*p* < 0.05).We found that no significant difference in the sham + EA group compared with the sham group. In addition, to discuss whether administering ML385 alone might lead to more severe stroke damage, we set up the MCAO/R + ML385 group, and from the results of behavioral and TTC staining, compared with the MCAO/R group, no significant statistical significance.

### EA Treatment Decreased Neuronal Damage and Ameliorated Neuronal Mitochondrial Injury after MCAO/R

**Fig. 3:**
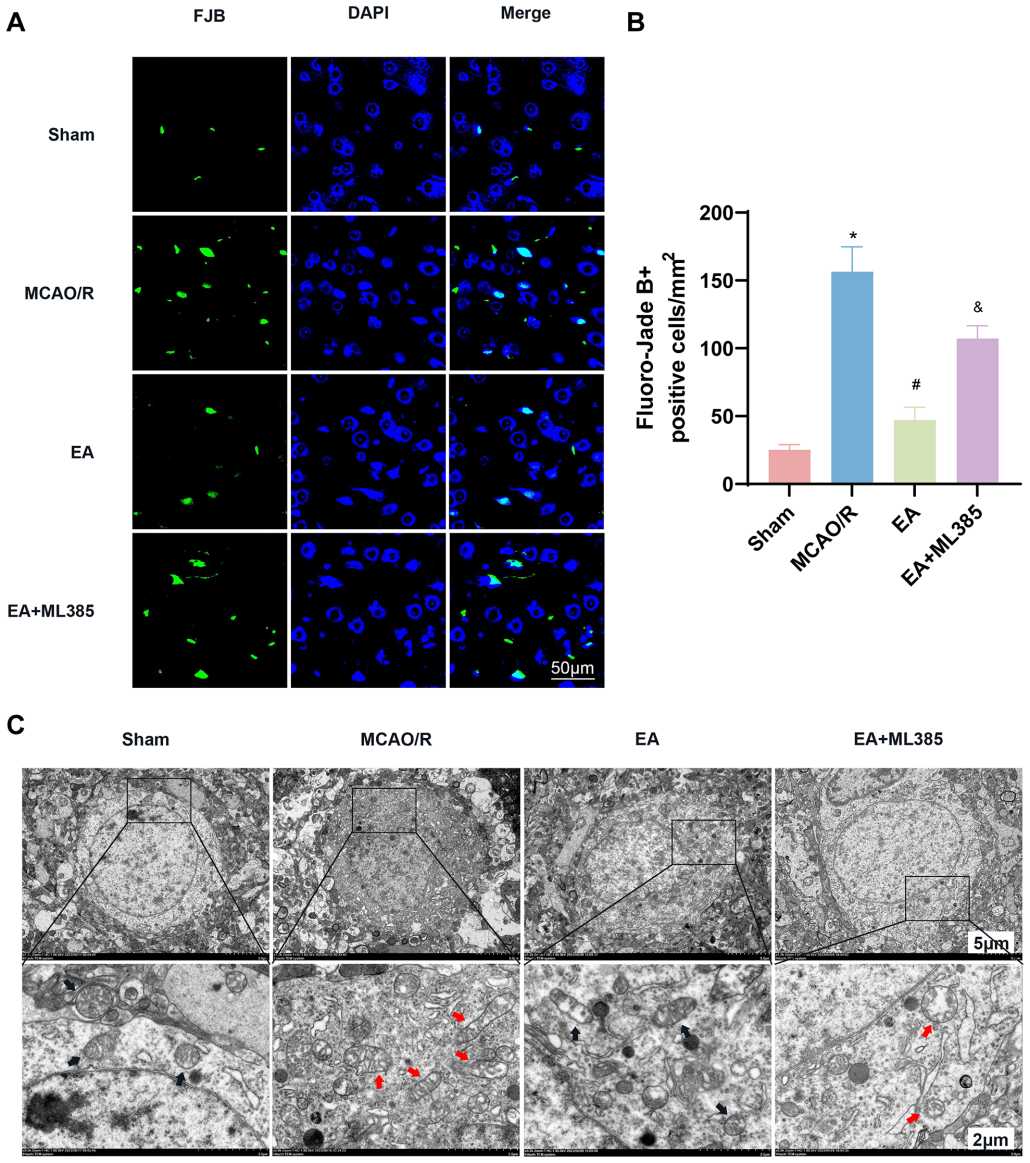
EA treatment alleviated the morphological and structural damage to cerebral cortex nerve cells after MCAO/R. (**A**) Representative fluorescence microscopy micrographs showing neurodegeneration, as assessed by Fluoro-Jade B+ (green) staining. (**B**) Quantitative analysis of Fluoro-Jade B + positive cells is shown (*n* = 6). (**C**) Mitochondrial morphological changes were detected by TEM. Black arrows indicate mitochondrial ultrastructure in the sham and EA groups, and red arrows indicate mitochondrial membrane rupture, cristae reduction, or disappearance in the MCAO/R and MCAO/R + ML385 groups

We observed neuronal damage in the ischemic cerebral cortex of rats following MCAO/R using Fluoro-Jade B staining. As illustrated in Fig. 3A and B, the MCAO/R group displayed a remarkable increase in the number of positive cells in comparison with the sham group. In contrast, Fluoro-Jade B fluorescence intensity decreased significantly in the EA group compared with the MCAO/R group. Furthermore, compared to the EA group, the EA + ML385 group showed an increase in the Fluoro-Jade B fluorescence intensity.

Electron microscopy revealed abnormal mitochondrial morphology, and increased membrane density, ruptured outer mitochondrial membranes, and a reduced number of cristae, which are characteristic of ferroptosis. As shown in Fig. 3C, transmission electron microscopy revealed intact outer cell membranes and abundant cristae in the sham group. The changes were typical of ferroptosis in the MCAO/R group compared to the sham group. EA group mitochondria were similar to sham group mitochondria, with a significant reversal of mitochondrial damage in the EA group. Mitochondrial damage was more severe in the MCAO/R + EA group compared to the EA group.

### EA Treatment Decreased the Level of Iron and Inhibited Oxidative Stress after MCAO/R

**Fig. 4:**
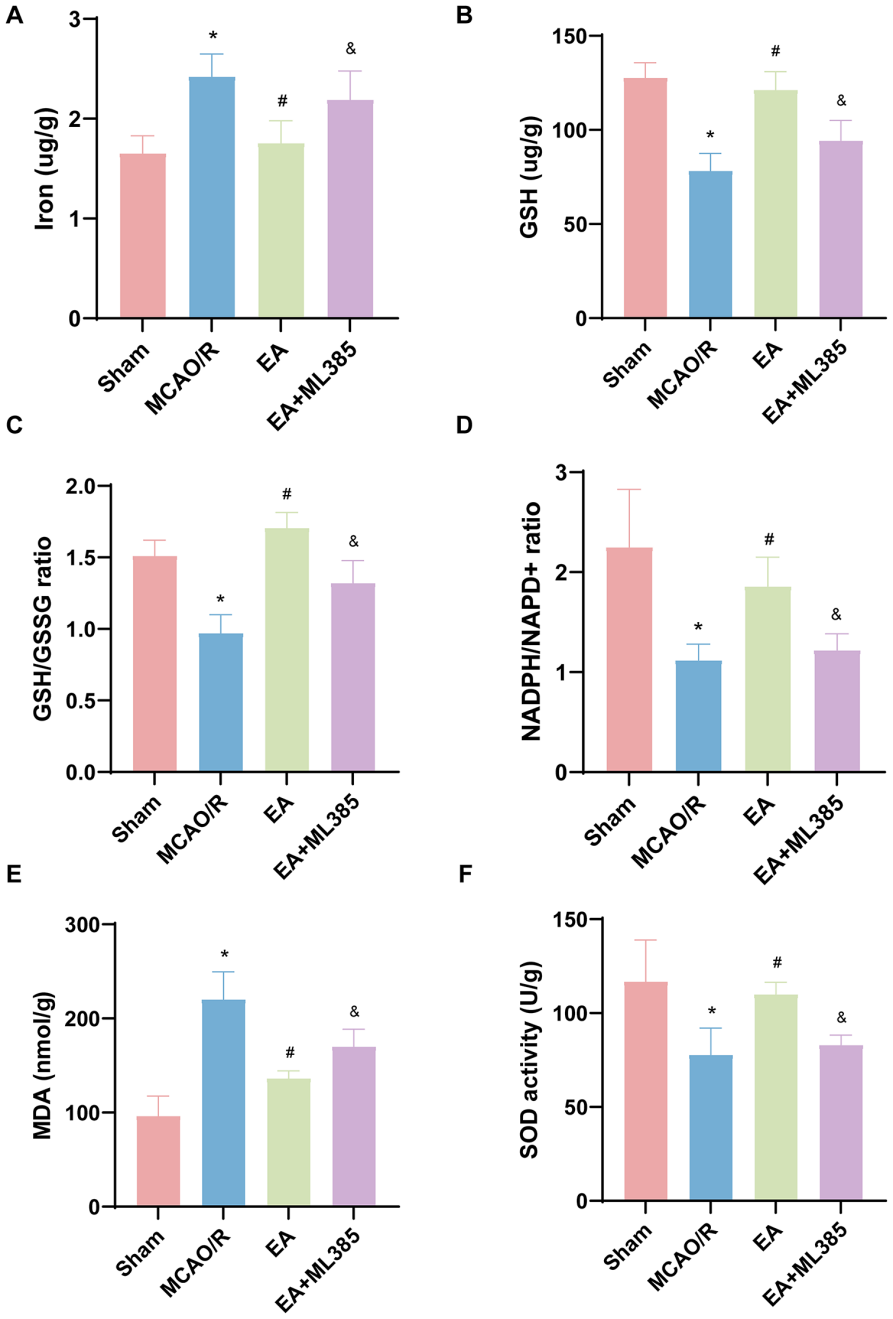
EA treatment reduced iron levels, improved lipid peroxidation and endogenous antioxidant levels after MCAO/R. Iron content (**A**) was investigated by ICP‒MS. GSH levels (**B**), the GSH/GSSG ratio (**C**), the NADPH/NADP + ratio (**D**), MDA levels (**E**) and SOD activity (**F**) were tested by ELISA. The results are expressed as means ± SD. *n* = 6. *p < 0.05 vs. the sham group; #*p* < 0.05 vs. the MCAO/R group; &*p* < 0.05 vs. the EA group

Ferroptosis is an iron-dependent form of cell death, and we analyzed tissue iron levels. As shown in Fig. 4A, significantly elevated iron levels were found after MCAO/R, and this increase in iron levels was blocked by EA (*p* < 0.05). Nonetheless, EA administration failed to reduce MCAO/R-induced iron accumulation after ML385 pretreatment (*p* < 0.05).

Increased lipid peroxidation activity led to ferroptosis, and GSH, NADPH, MDA, and SOD were used as antioxidant markers. The effect of EA on GSH, MDA, and SOD activities and the GSH/GSSG and NADPH/NADP + ratios was then examined. As shown in Fig. 4B-F, MCAO/R significantly decreased GSH levels and SOD activities and the GSH/GSSG and NADPH/NADP + ratios in the brain (*p* < 0.05) and increased MDA levels (*p* < 0.05), and these changes were reversed by EA (*p* < 0.05). The groups EA + ML385 and MCAO/R had comparable levels of GSSH, SOD, and MDA as well as GSH/GSSG and NADPH/NADP + ratios. In the EA + ML385 group, MDA content in the brain tissue increased compared to the EA group, while the other indicators decreased significantly (*p* < 0.05).

### EA Treatment Regulated the Expression of Ferroptosis-Related Proteins after MCAO/R

**Fig. 5:**
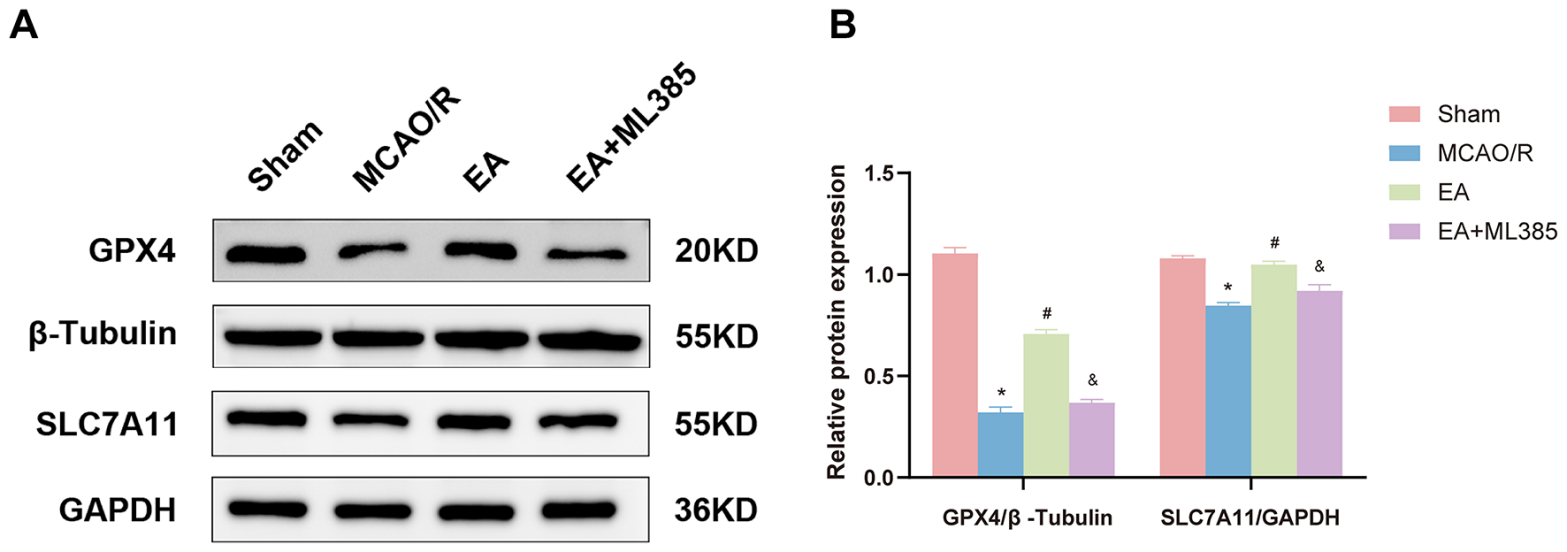
EA treatment increased the expression of ferroptosis-related protein levels after MCAO/R. **(A, B)** GPX4 protein levels were measured by Western blotting. β-Tubulin was used as a loading control. SLC7A11 protein levels were measured by Western blotting. GAPDH was used as a loading control. The Western blot image is representative of three independent experiments. The results are expressed as means ± SD. *n* = 6. *p < 0.05 vs. the shame group; #*p* < 0.05 vs. the MCAO/R group; &*p* < 0.05 vs. the EA group

GPX4 is a key marker of ferroptosis, and GSH is an essential cofactor for it. SLC7A11 serves as the principal cysteine source for GSH synthesis [40, 41], and Nrf2 can impede ferroptosis by controlling SLC7A11 expression [22, 24, 42]. We employed Western blotting to assess the expression of GPX4 and SLC7A11, two ferroptosis-related proteins, in rat brain tissue. The study results (Fig. 5) suggest that the MCAO/R group had lower GPX4 and SLC7A11 protein expression levels in brain tissue than the sham group (*p* < 0.05). Conversely, EA significantly enhanced the protein expression of GPX4 and SLC7A11 (*p* < 0.05). Furthermore, the Nrf2 inhibitor ML385 effectively counteracted the impact of EA on GPX4 and SLC7A11 protein levels in rat brain tissue post-MCAO/R (*p* < 0.05).

### EA Treatment Facilitated Nrf2 Activation after MCAO/R

**Fig. 6:**
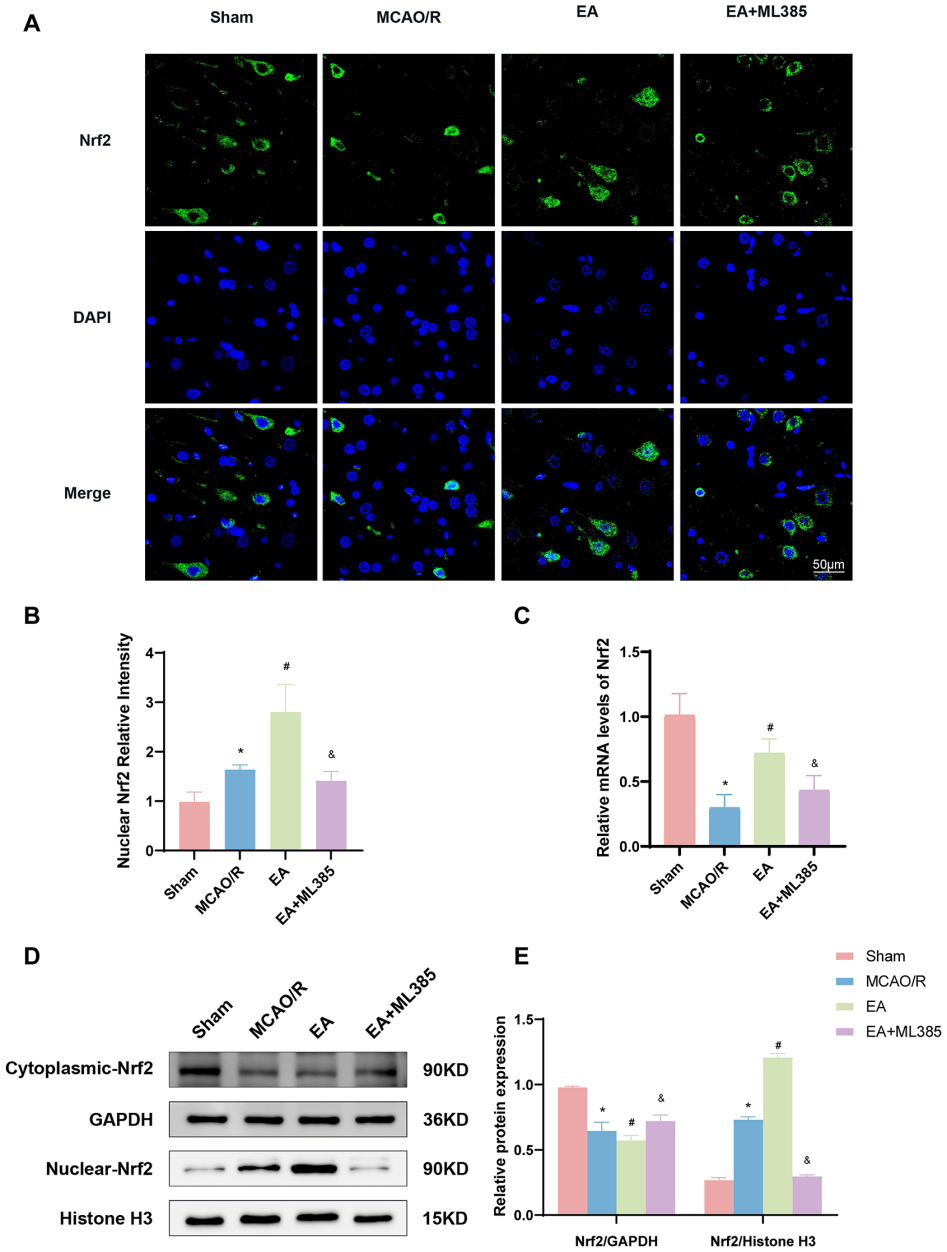
EA treatment promoted Nrf2 activation after MCAO/R. **(A)** Immunofluorescence images of nuclear Nrf2 in the cerebral cortex nerve cells. **(B)** Quantitative analysis of the fluorescence intensity of nuclear Nrf2. **(C)** Levels of Nrf2 mRNA in each group. **(D, E)** Cytoplasmic Nrf2 and nuclear Nrf2 protein levels were measured by Western blotting. Histone-H3 was used as a loading control for nuclear fractions. Data were averaged from three independent experiments. The results are expressed as means ± SD. *n* = 6. *p < 0.05 vs. the sham group; #*p* < 0.05 vs. the MCAO/R group; &*p* < 0.05 vs. the EA group

We measured Nrf2 levels in the ischemic cerebral cortex cells of rats through immunofluorescence staining experiments. As illustrated in Fig. 6A and B, Nrf2 was present in the cytoplasm in the sham group. In contrast, in the MCAO/R group, Nrf2 fluorescence intensity increased in the nucleus in comparison with the sham group. Furthermore, the fluorescence intensity of Nrf2 in the nucleus was markedly higher in the EA group as compared to the MCAO/R group. It was observed that the fluorescence intensity of Nrf2 in the nucleus was reduced in the EA + ML385 group as compared to the EA group.

Next, we utilized RT‒qPCR to evaluate Nrf2 mRNA expression and WB to assess Nrf2 protein expression in rat brain tissue. The data indicated a noteworthy decrease in Nrf2 mRNA levels in rat brain tissues in the MCAO/R group, which was reversed by EA (*p* < 0.05, Fig. 6C). Furthermore, MCAO/R injury slightly elevated nuclear Nrf2 levels and reduced cytoplasmic Nrf2 levels (*p* < 0.05, Fig. 6D and E). However, EA significantly increased the protein level of nuclear Nrf2 and decreased the level of cytoplasmic Nrf2 (*p* < 0.05). In contrast, the ML385, the Nrf2 inhibitor, significantly reversed the influence of EA on Nrf2 protein and Nrf2 mRNA levels in rat brain tissue after MCAO/R (*p* < 0.05).

## Discussion

The pathological damage of cerebral ischemia and reperfusion is based on the occurrence of ischemia and hypoxia in neuronal cells at the site of blood supply obstruction, during which apoptosis, necroptosis, autophagy, and oxidative stress are involved in the damage process [43]. EA is a therapeutic method that combines traditional acupuncture with modern electrical stimulation. It can protect neurons and improve neurological function after cerebral ischemia through multiple pathways. In this present study, we demonstrates that rats treated with EA had significantly lower neurological impairment, cerebral infarct volume and neuronal injury compared to rats that did not undergo the EA treatment.

Ferroptosis, a novel type of programmed cell death different from apoptosis, is characterized by the accumulation of lipid-reactive oxygen species and an increase in intracellular iron content [9]. Ferroptosis is characterized by mitochondrial shrinkage, an increased membrane density, and a reduced number or loss of cristae [8]. Numerous studies have confirmed that ferroptosis contributes to the onset and progression of various human diseases. These include, but are not limited to, tumors [44, 45], cardiovascular diseases [46], urological diseases [47], and neurodegenerative diseases [48]. Additionally, ferroptosis plays a crucial role in the development of IS and cerebral ischemia-reperfusion injury. The present study examined the levels of MDA, SOD, GSH, and NADPH, which are the main oxidative stress biomarkers. The results showed that EA treatment significantly increased SOD activity, total GSH levels, GSH/GSSG and NADPH/NADP + ratios, while decreasing MDA levels. Additionally, in the MCAO/R model, mitochondria exhibited ferroptosis signature, while EA treatment had a protective effect on mitochondrial structure. Furthermore, EA treatment results in a reduction in tissue iron levels. This is in line with the findings of our prior experiments.

**Fig. 7:**
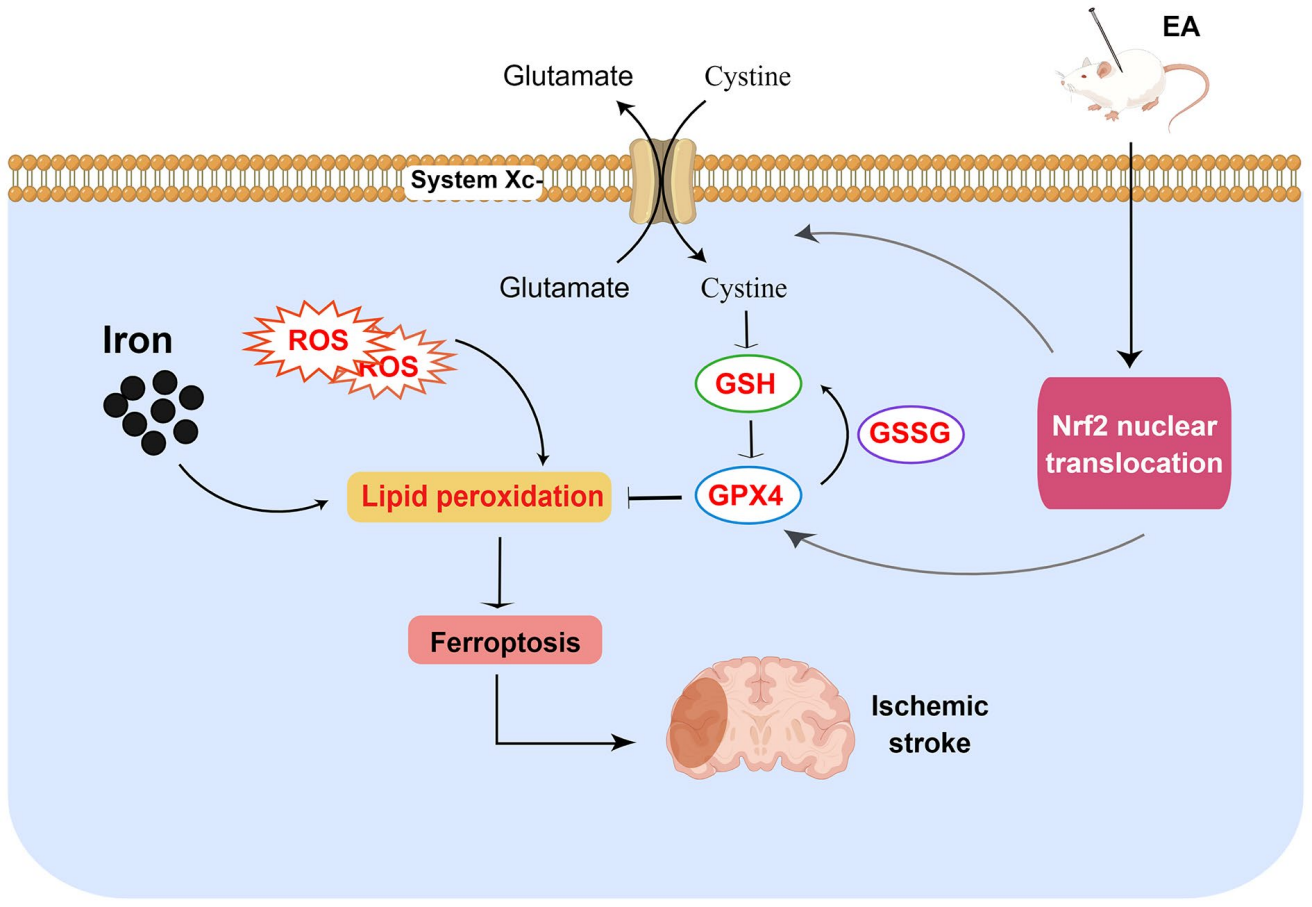
Schematic diagram showing the protective mechanism on ischemic stroke by which EA decreases ferroptosis by activating Nrf2. In cases of cerebral ischemia/reperfusion, EA can alleviate injury by inhibiting ferroptosis through the promotion of Nrf2 expression and nuclear translocation, regulating SLC7A11 and GPX4 expression, inhibiting lipid peroxidation, and increasing endogenous antioxidant activity

Nrf2 is a crucial transcription factor that regulates cellular redox equilibrium and inflammatory responses [26, 27] and is involved in regulating the ferroptosis cascade [24]. Nrf2 is localized in the cytoplasm and is activated in response to cellular stimulation [49]. Meanwhile, Nrf2 serves as a transcription factor and can regulate the expression of SLC7A11 and GPX4 to inhibit lipid peroxidation and ferroptosis [24]. Pan et al. demonstrate that activating the Nrf2/HO-1 pathway and inhibiting oxidative stress can protect the brain from I/R damage [50]. Thus, we utilized WB to measure the levels of Nrf2, GPX4, and SLC7A11 and RT‒qPCR to assess Nrf2 mRNA expression. Our findings indicate that EA increased Nrf2 mRNA expression and the levels of nuclear Nrf2 protein, GPX4, and SLC7A11 protein in MCAO/R rat brain tissue. Meanwhile, EA reduced Nrf2 cytoplasmic protein levels. We also observed that EA promoted Nrf2 nuclear translocation, as supported by the immunofluorescence results. Interestingly, the Nrf2 inhibitor ML385 reversed the effects of EA. These results (Fig. 7) indicate that ferroptosis significantly contributes to cerebral ischemia‒reperfusion injury and that EA may inhibit ferroptosis associated with IS by activating Nrf2 and upregulating SLC7A11 and GPX4.

## Conclusion

In summary, this study enhances our knowledge of how EA guards against cerebral ischemia and reperfusion injury and uncovers the potential mechanisms of action of EA. Initially, we demonstrated the occurrence of ferroptosis in a cerebral ischemia/reperfusion rat model. Additionally, electroacupuncture was shown to decrease the volume of cerebral infarct and promote neurological function in cerebral ischemia/reperfusion rats. Second, electroacupuncture was found to potentially alleviate cerebral ischemia/reperfusion injury by hindering ferroptosis through various mechanisms, such as by inhibiting lipid peroxidation, increasing endogenous antioxidant activity, and altering the expression of Nrf2 and ferroptosis-related proteins.

## Data Availability

The data utilized to bolster the results of this research are incorporated within the article.
